# Washoff of cypermethrin residues from slabs of external building material surfaces using simulated rainfall

**DOI:** 10.1002/etc.2432

**Published:** 2013-10-15

**Authors:** Jennifer R Trask, Christopher M Harbourt, Paul Miller, Megan Cox, Russell Jones, Paul Hendley, Chung Lam

**Affiliations:** † Waterborne Environmental, LeesburgVirginia, USA; ‡ Bayer CropScience, Research Triangle ParkNorth Carolina, USA; § Syngenta Crop Protection, GreensboroNorth Carolina, USA

**Keywords:** Washoff, Pyrethroid, Simulated rainfall, Building material

## Abstract

The use of pesticides by homeowners or pest-control operators in urban settings is common, yet contributions of washoff from these materials are not easily understood. In the present study, cypermethrin, formulated as Cynoff EC (emulsifiable concentrate) and Cynoff WP (wettable powder) insecticides, was applied at typical rates to 10 different building material surfaces to examine its washoff potential from each surface. Using an indoor rainfall simulator, a 1-h rainfall event was generated and washoff samples were collected from 3 replicates of each surface type. Washoff was analyzed for cypermethrin using gas chromatography-negative chemical ionization mass spectrometry. An analysis of variance for a split-plot design was performed. Many building materials had similar water runoff masses, but asphalt resulted in significantly reduced average water runoff masses (73% less). The Cynoff WP formulation generally produced greater cypermethrin washoff than the Cynoff EC formulation. In addition, results for both the WP and EC formulations indicated that smoother surfaces such as vinyl and aluminum siding had higher washoff (1.0–14.1% mean percentage of applied mass). Cypermethrin washoff from rough absorptive surfaces like concrete and stucco was lower and ranged from 0.1 to 1.3% and from 0 to 0.2%, respectively, mean percentage of applied mass. Both building material surface and formulation play a significant role in cypermethrin washoff. *Environ Toxicol Chem* 2014;33:302–307. © 2013 The Authors. *Environmental Toxicology and Chemistry* published by Wiley Periodicals, Inc. on behalf of SETAC. This is an open access article under the terms of the Creative Commons Attribution License, which permits use, distribution, and reproduction in any medium, provided the original work is properly cited.

## INTRODUCTION

The use of pyrethroids has increased since the 1990s as a result of the phase-out of the organophosphate insecticides in both the agricultural and urban environments. In California, USA, for example, pyrethroid sales for use in agricultural and urban areas increased from 2000 to 2006 according to the California Department of Pesticide Regulation, although sales in California have shown a downward trend since 2007 1. Recent studies have documented detection of pyrethroids in various stream environments across the United States 2–5. These pesticides are commonly used by professionals and homeowners for treatments around structures (e.g., house foundations), perimeters, and landscapes. In California, >95% of the reported urban use for major pyrethroid chemicals is for structural pest control 1. Work performed by Greenberg et al. 6 has shown pyrethroid residues moving to the street in runoff as a result of applications around the border of residential structures. Weston and Lydy 2 also found pyrethroid residues in runoff entering various California urban streams; however, specific sources were not identified. Given their wide distribution of use, specific sources of pyrethroids that have the potential to contribute residues to urban streams have been a challenge to characterize.

Although sources of residues from pyrethroids are fairly well understood in agricultural environments, knowledge about urban sources (i.e., urban landscapes) that may contribute pyrethroid residues to nearby water bodies is limited. Few studies have focused on hardscape materials found in urban areas and examined the potential impact of runoff from these materials. Weston et al. 3 found pyrethroid residues in runoff from residential neighborhoods around Sacramento, California, USA. Although the source was thought to be primarily professional pest controllers, they concluded that use by homeowners may also contribute. Jorgenson and Young 7 and Jorgenson et al. 8 examined washoff from concrete surfaces and concluded that washoff is a function of the product formulation. A study by Jiang et al. 9 examined the effects of time on desorption following application of 4 pyrethroids to small concrete blocks in the laboratory. They concluded that formulation and persistence of the chemicals on the concrete surface over time may be a factor in washoff potential. In another study by Jiang et al. 10, 2 pyrethroids were tested under field conditions in California and demonstrated similar behaviors as observed in the laboratory study. They also investigated the effects of surface conditions (i.e., sealing, acid washing) on potential runoff of pyrethroids and found only minor differences yet suggested that surface roughness may play a role in reducing pyrethroid transport.

The purpose of the present study was to determine differences in washoff of cypermethrin applied to various building material surfaces. The present study also included 2 classes of residential insecticide formulations (emulsifiable concentrate [EC] and wettable powder [WP]) to compare washoff between formulations.

## EXPERIMENTAL SECTION

### Test materials

The building materials selected include those found on surface areas of structures (e.g., driveways, foundations) that may receive applications of pyrethroids. In the present study, 6 different building materials of varying surface finishes were tested. These included clean unpainted and painted concrete, clean unpainted and painted stucco, clean aluminum siding, clean vinyl siding, clean asphalt, and clean unpainted and painted wood. A dusty painted wood surface was also chosen to see if particulate materials affect pyrethroid washoff. Each test slab was approximately 23 cm × 61 cm (nominal 9 in × 24 in), but of varying thicknesses (∼3.9–11.4 cm) depending upon the material. Latex exterior paint was used on all painted surfaces. Additional slabs made of clean aluminum siding were used as field blanks (i.e., untreated slabs). For each building material surface (e.g., painted concrete), 6 slabs were constructed to provide 3 replicates per pyrethroid formulation.

Prior to application, each building material surface was washed and rinsed, with the exception of dusty painted wood. Unpainted concrete slabs were soaked for at least 2 d to remove any substances from the surface that might affect the pH. This was important for the concrete slabs because freshly poured concrete can have a pH greater than 10 11, and pyrethroids can hydrolyze more quickly under alkaline conditions 7,12, which might have biased results for substrates that can generate local high pH environments (e.g., concrete). Small amounts of muriatic acid (31.45% hydrochloric acid) were added each day to the water during the soaking process to aid in neutralizing the surface of the unpainted concrete slabs. Dusty painted wood surfaces were created by pouring and rubbing soil (obtained from the Central Valley of California) onto the painted surface after it was cleaned, rinsed, and dried. The soil obtained was analyzed for cypermethrin, which was <0.1 µg/L. Municipal water from the city of Urbana, Illinois, USA, was used for washing, soaking, and rainfall simulation events, which had a pH between 8.5 and 9.0 (Illinois American Water, personal communication). More information regarding the test materials is available in the Supplemental Data.

### Test substance application

Two commercial cypermethrin formulations were selected for the present study: Cynoff EC insecticide (24.8% cypermethrin) and Cynoff WP insecticide (40% cypermethrin). Certified test materials were obtained from FMC Corporation and stored at room temperature. The 60 building material test slabs were divided into 6 groups of 10, with 3 replicate groups per formulation. Cypermethrin was applied using a laboratory track sprayer at the University of Illinois at Urbana-Champaign in Urbana, Illinois, USA (Supplemental Data, Figure S1). The system was calibrated to make applications to the test slab area at the recommended maximum label rate. This corresponded to 26.5 mg and 28.1 mg active ingredient (a.i.) per slab for Cynoff EC and Cynoff WP insecticides, respectively.

To quantify the mass of test substance being applied to the slabs, application monitoring samples (filter paper samples) were collected every fifth application, resulting in 2 filter paper samples per replicate group. Samples were collected using 15-cm–inner diameter glass Petri dishes, the lids of which were fitted with Whatman 15-cm-diameter filter paper.

Immediately following applications, test slabs were removed from the booth and placed into individual opaque storage containers for drying and storage until rainfall simulation. Additional details about the track sprayer equipment and application are available in the Supplemental Data.

### Simulated rainfall events

Simulated rainfall events were conducted using a 3-story indoor laboratory rainfall simulator at the University of Illinois. The simulator contains 2 emitter modules, each containing 5 oscillating nozzles located 10 m above the test floor. This distance allows the simulated raindrops to attain terminal velocity upon impact with the simulator floor, with a droplet size, speed, and energy representative of natural rainfall 13. The rainfall simulator's uniform test floor is 1.82 m × 4.65 m (6 ft × 15 ft), making room for 11 test slab locations during a rainfall event. Details regarding the mechanics and operations of the simulator have been well documented ([13]; J. R. Trask, 2002, Master's thesis, University of Illinois at Urbana-Champaign, Urbana, IL, USA).

Each replicate group was subjected to a single rainfall event for 1 h at a rate of 2.54 cm/h (1 in/h). A field blank was included with each rainfall event. A test stand was constructed to hold the test slabs at a 60-degree angle from the horizontal. This angle was chosen to simulate wind causing rainfall to impact a vertical surface at a similar angle in the environment 14,15. Stainless steel flashing and collection devices were attached to the sides and the downslope face of the slab, respectively (Supplemental Data, Figure S2). The design ensured that rainwater flowed down the length of the slab surface and into the collection device while minimizing losses to splashing. Aluminum rain shields were attached to the top of the collection device to prevent any direct rainfall (i.e., rainfall not in contact with the test slab) from being collected in the sample bottle. Runoff from each slab was collected in a precleaned 2-L amber glass bottle and preserved with sufficient 10% formic acid to lower the pH between 5 and 7, maximizing pyrethroid chemical stability. Rainfall simulation timings were approximately 24 h after test substance application. Additional details regarding sample collection and test slab placement are provided in the Supplemental Data.

### Chemical analysis

Sample analyses were performed at Bayer CropScience laboratories in Stilwell, Kansas, USA. A validated analytical method was used to analyze the following matrices: water samples, emptied bottles, filter papers, and spray solutions. The method employed a liquid–liquid partition followed by gas chromatography with negative chemical ionization mass spectrometric detection. The limit of quantitation in water as specified for the method was 1.03 µg/L. This limit of quantification value represents 0.01% of applied cypermethrin (assumes an average sample volume of 1.7 L and a target mass applied of cypermethrin as Cynoff EC insecticide of approximately 26.5 mg a.i. per slab or approximately 28.1 mg a.i. per slab as Cynoff WP insecticide, based on product label information). Although recent studies involving pyrethroids have used limits of quantification as low as 1 part per trillion 16, the limits of quantification set for these analyses are adequate considering the application rates and the range of concentrations determined in the washoff samples. Calibration standard solutions were prepared using a primary stock standard of cypermethrin (purity 93.9%) and an internal standard of cyfluthrin-methyl-d6 dissolved in acetonitrile (standard K-939, 16% by wt). A 5-point standard calibration curve was prepared as described in the method and consisted of the following amounts: 7.23 µg/L, 20.7 µg/L, 51.7 µg/L, 103 µg/L, and 207 µg/L. This calibration curve range (7.23–207 µg/L) was equivalent to a sample concentration range of 0.36 µg/L to 10.4 µg/L in the washoff samples. Procedural recoveries at a concentration of 10.3 µg/L were run with each set of analyses, and the average recovery of the 9 procedural recovery samples was 92% with a relative standard deviation of 1%. Prior to extraction, runoff samples were allowed to come to room temperature, thoroughly agitated, and then analyzed for cypermethrin in water. Other matrices analyzed were the empty bottles from the washoff samples, the tank mix samples, and the application monitoring samples (filter papers). The empty sample bottles were washed with dichloromethane, and then the rinsate was analyzed to determine the amount of cypermethrin remaining in the sample bottle. The estimated mass of cypermethrin recovered from the empty bottle was added to the amount of cypermethrin found in the water phase of the washoff sample. Application monitoring samples were extracted and washed with an acetonitrile/acetone solution (2:98 by volume) and then analyzed for cypermethrin. More information about sample extraction can be found in the Supplemental Data.

### Data analysis

An analysis of variance (ANOVA) for a split-plot design was performed using the Statistical Analysis Software package (SAS Institute) 17 to investigate the following hypotheses: there is no difference in pyrethroid washoff from different building materials, and there is no difference in pyrethroid washoff between different formulations of cypermethrin. This mixed-model analysis was used to determine whether or not building material, formulation, and the interaction between these variables were significant contributors in the model for water runoff mass and the percentage of washoff of cypermethrin. Tukey's multiple comparison adjustment was used to determine significant differences (α* *< 0.05) between the building materials and the 2 formulation types.

The mechanics of the rainfall simulator limited the number of slabs tested in a single experimental simulated event. In addition, applications had to be grouped by formulation to 1) comply with the application-to-rainfall time constraint, and 2) avoid possible cross-contamination. The split-plot design made it possible to evaluate the influence of the main effects, building material and formulation in regard to the mass of water runoff, and the percentage of washoff of cypermethrin and to evaluate the interaction of the factors. The simulation event was also included in the model. The fixed part of the model consisted of the following form to evaluate the main effects and their interaction: formulation + building material + (formulation × building material). The random part of the model was the interaction of formulation and simulation event. This analysis was followed by comparing differences of means using the Tukey adjustment.

## RESULTS

### Application monitoring samples

The application monitoring samples were used to determine the mean mass of cypermethrin applied to the test slabs for each formulation. Using the effective spray interception area per Petri dish (176.4 cm^2^/dish) and the effective spray interception area per building material slab (1393.5 cm^2^/slab), the mean mass recovered was determined for each formulation. The recovered mean mass for Cynoff EC insecticide from the Petri dishes, as measured from the laboratory analysis, was 4.27 ± 0.11 mg a.i. (*n *= 6), while the mean mass recovered for Cynoff WP insecticide was 5.40 ± 0.52 mg a.i. (*n* = 6). By scaling these masses based on the relative areas of Petri dishes and slabs, the spray solution loadings per slab were 33.8 mg a.i. and 42.7 mg a.i. for the EC insecticides and WP insecticides, respectively. The masses per Petri dish recovered demonstrate that the cypermethrin application in the present study was higher than the target rates. The final washoff results were based on these estimated mean masses of cypermethrin applied (as opposed to the target values) to the building material slabs for each formulation.

### Simulated rainfall and runoff

The rainfall simulator performed consistently with little variability between simulation events. The mean rainfall amount for all events was within 10% of the target rainfall amount (2.54 cm). The mean water runoff masses varied from 468.3 g for asphalt to 1988.9 g for clean unpainted concrete (Supplemental Data, Figure S4). The mean runoff masses were used with the mean rainfall to determine runoff coefficients from each surface; the runoff coefficients ranged between 0.80 to 1.06 with the exception of the asphalt, which was estimated to be approximately 0.25. Typical values for impervious surfaces such as concrete and asphalt range from 0.70 to 0.95 18, while urban playground and suburban areas are between 0.20 and 0.40 18. Clean painted and unpainted concrete and dirty painted wood had runoff coefficients >1; however, the positions of these slabs within the simulator most likely received higher rainfall than the mean rainfall, giving a runoff coefficient closer to 1.0.

The split-plot analysis using SAS software (mixed-models procedure) 17 was used to determine if there were significant differences in the collection of runoff between building materials and formulations. The analysis showed that formulation was not a significant factor. There were some differences among building materials in water runoff mass (mean range 1505–1989 g), but asphalt (mean 468 g) was found to be significantly different from all other building materials (*p *< 0.05).

The masses for the asphalt slabs ranged between 295 g and 591 g. The low recovered water masses are most likely a result of the material absorbing water during the simulation events. This may be related to the level of compaction of the material during the construction phase or the lack of a sealant, which is typically used on roads to prevent water absorption over time. In addition, there may have been splash or leakage from the test slabs, which contributed to a lower water runoff mass; however, none of these potential causes were observed. Additional information regarding rainfall, water runoff, and transit stability results can be found in the Supplemental Data.

### Building material slab cypermethrin washoff results

**Figure 1:**
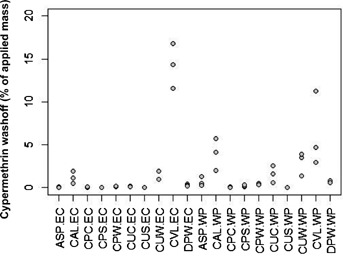
Washoff of cypermethrin as percentage of amount applied for 10 building materials and by formulation (*n *= 3 per building material/formulation). ASP = clean asphalt; CPW = clean painted wood; CAL = clean aluminum siding; CPS = clean painted stucco; CVL = clean vinyl siding; DPW = dirty painted wood; CPC = clean painted concrete; CUS = clean unpainted stucco; CUC = clean unpainted concrete; CUW = clean unpainted wood; EC = emulsifiable concentrate; WP = wettable powder.

Masses of cypermethrin in runoff water from the building material slabs were added to the masses recovered from the walls of the washoff sample container. The total mass of cypermethrin that washed off each building material slab was compared with the estimated mean applied mass for each formulation (determined from the application monitoring samples). Data are presented graphically (Figure 1) in terms of percentage of applied cypermethrin for all building materials and formulations. Field blanks (clean aluminum siding) were analyzed, and the runoff contained no detectable residues (less than limit of quantification of 1.03 µg/L).

The results showed that clean vinyl siding produced the highest mean percentage of washoff, while clean unpainted stucco generated the lowest values for both formulations. With the exception of clean vinyl siding, the Cynoff WP formulation washoff values were higher than for the EC formulation as percentage of applied cypermethrin. The washoff data produced residuals that were not normally distributed, so the data were log-transformed, which produced residuals that satisfied the assumptions of normality and equal variance. Tables1 and 2 present the washoff means expressed as a percentage of applied cypermethrin for the EC and WP formulations, respectively. The analysis showed that building material, formulation, and their interaction were significant. Building material and formulation (main effects) were most significant (*F* = 82.53 and 76.64, respectively), while the interaction was <10% of the main effect *F* values. Therefore, it is reasonable to say that 1 formulation had a far higher percentage overall and the building materials surface did impact cypermethrin washoff.

**Table 1:** Cypermethrin washoff of applied mass (means and 95% confidence intervals) and summary of statistical significance (Tukey's test) for Cynoff EC formulation

Building material	Cynoff EC insecticide washoff (% of applied mass)	Tukey's grouping^a^
Clean vinyl siding	14.1 (7.7–25.8)	A
Clean unpainted wood	1.6 (0.85–2.86)	B
Clean aluminum siding	1.0 (0.57–1.9)	B,C
Dirty painted wood	0.30 (0.16–0.55)	C,D
Clean unpainted concrete	0.14 (0.07–0.25)	D,E
Clean painted wood	0.10 (0.06–0.19)	D,E
Clean asphalt	0.06 (0.03–0.12)	D,E
Clean painted concrete	0.06 (0.03–0.12)	D,E
Clean painted stucco	0.04 (0.02–0.07)	E
Clean unpainted stucco	<0.01 (0.00–0.01)	F

a Tukey's multiple comparison test evaluates pairwise differences between materials. Materials sharing the same letter have mean percentage ofwashoffs of applied mass that are not significantly different. For example, clean painted stucco for the EC is an “E,” which means there is no difference between it and other “E” designations such as clean painted concrete; however, clean vinyl siding is an “A,” which means there is a significant difference between it and clean painted stucco.

EC = emulsifiable concentrate.

**Table 2:** Cypermethrin washoff of applied mass (means and confidence intervals) and summary of statistical significance (Tukey's test) for Cynoff WP formulation

Building material	Cynoff WP insecticide washoff (% of applied mass)	Tukey's grouping^a^
Clean vinyl siding	5.4 (2.9–9.9)	A
Clean aluminum siding	3.6 (2.0–6.6)	A,B
Clean unpainted wood	2.7 (1.5–4.9)	A,B,C
Clean unpainted concrete	1.3 (0.73–2.4)	B,C,D
Dirty painted wood	0.65 (0.35–1.2)	C,D,E
Clean asphalt	0.57 (0.31–1.04)	C,D,E
Clean painted wood	0.42 (0.23–0.77)	D,E
Clean painted stucco	0.22 (0.12–0.41)	E,F
Clean painted concrete	0.08 (0.04–0.14)	F,G
Clean unpainted stucco	0.04 (0.02–0.07)	G

a Tukey's multiple comparison test evaluates pairwise differences between materials. Materials sharing the same letter have mean percent washoffs of applied mass that are not significantly different. For example,clean painted stucco for the WP is an “E,” which means there is no difference between it and other “E” designations such as clean painted wood; however, clean vinyl siding is an “A,” which means there is a significant difference between it and clean painted stucco.

WP = wettable powder.

Tukey's adjustment was used to examine which building materials were significantly different from each formulation. For the EC formulation, many building material surfaces proved to be significantly different (from each other; *p *< 0.05), which can be seen in Table1. Clean vinyl siding (14.1%) and clean unpainted stucco (<0.01%) were significantly different from all other building materials, representing the highest and lowest percentage of applied washoff, respectively; the remaining materials were distributed into 3 additional groups. Materials with smoother surfaces such as clean unpainted wood (1.6%) and clean aluminum siding (1.04%) were found to be significantly different from textured surfaces such as clean unpainted concrete (0.14%). Dirty painted wood (0.30%) was found to be similar to most materials excluding clean vinyl siding, clean unpainted wood, and clean painted and unpainted stucco. Smoother surfaces produced higher washoff values than textured surfaces when comparing individual building material means.

Washoff results from the WP formulation, unlike the EC formulation, showed there was no single building material that was significantly different from all others. As seen in Table2, clean vinyl siding (5.4%) was not significantly different from clean aluminum siding (3.6%) and clean unpainted wood (2.7%) but was different from all other building materials. Clean unpainted stucco (0.04%) was found to be significantly different from all building materials except clean painted concrete (0.08%). The remaining materials showed more similar washoff fractions than the EC formulation. For example, clean aluminum siding was found to be similar to clean vinyl siding, clean unpainted wood, and clean unpainted concrete but significantly different from all others. Dirty painted wood (0.65%) was significantly different from clean vinyl siding, clean aluminum, clean painted concrete, and clean unpainted stucco. Similar results were seen when the comparison of building materials independent of formulation was examined (Table3). The study suggested that the effects of surface roughness played a significant role in the losses of cypermethrin from building material surfaces.

**Table 3:** Cypermethrin washoff of applied mass (means and confidence intervals) and summary of statistical significance (Tukey's test) of building materials (*n* = 6)

Building material	Cypermethrin washoff (% of applied mass)^a^	Tukey's grouping^b^
Clean vinyl siding	8.7 (5.7–13.4)	A
Clean unpainted wood	2.0 (1.3–3.1)	B
Clean aluminum siding	1.9 (1.3–3.0)	B
Dirty painted wood	0.44 (0.29–0.68)	C
Clean unpainted concrete	0.42 (0.28–0.66)	C
Clean painted wood	0.21 (0.14–0.32)	C,D
Clean asphalt	0.19 (0.12–0.30)	C,D,E
Clean painted stucco	0.09 (0.06–0.14)	D,E
Clean painted concrete	0.07 (0.05–0.11)	E
Clean unpainted stucco	0.01 (0.01–0.02)	F

a Analysis includes both formulations (EC and WP) in a single group per building material surface,which are compared.

b Tukey's multiple comparison test evaluates pairwise differences between materials. Materials sharing the same letter have mean percent washoffs of applied mass that are not significantly different. For example,clean painted concrete is an “E,” which means there is no difference between it and other “E” designations such as clean painted stucco; however, clean vinyl siding is an “A,” which means there is a significant difference between it and clean painted concrete.

EC = emulsifiable concentrate; WP = wettable powder.

The analysis also showed that formulation was a significant factor in the percentage of washoff. The ANOVA results showed overall that the EC formulation (0.19%) behaved differently from the WP formulation (0.61%). Although the mean percentage of washoff values, when examining individual building materials, showed that greater losses occurred from the WP formulation than the EC formulation except for clean vinyl siding, only 4 building materials showed significant differences (*p *< 0.05) in washoff percentage between the EC and WP formulations, based on Tukey's adjusted *p* values. These building materials—asphalt, clean painted and unpainted stucco, and clean unpainted concrete—had lower washoff than most of the other building materials, excluding clean unpainted concrete. This suggested, in general, that as the surface of the material became smoother there was less impact of formulation on the resulting washoff characteristics from the treated building material surfaces. For example, clean painted concrete was the least significant of the materials in terms of formulation differences (*p* = 1.0), followed by clean unpainted wood (*p *= 0.99). Clean vinyl siding (*p *= 0.74) produced the highest percentage of applied washoff (Table3) and was significant when comparing building materials, but it was not significantly different in terms of formulation. This may be because of the particle size of the applied formulation versus the available surface area coverage with respect to the depth of coverage. It may also be a function of the final prepared solution and particle size (i.e., colloidal vs dissolved particle). The track sprayer was able to deliver a uniform application, so it is unlikely that the differences were caused by differences in application techniques. The hypothesis that there is no difference in pyrethroid washoff between different formulations of cypermethrin was rejected based on the analysis. However, one must consider that the results for the WP formulation exhibited increased variability (as measured by the nontransformed data standard deviation) between replicates compared with the same building material surfaces with the EC formulation. The present study demonstrates that the type and texture of the building material surface in combination with the formulation applied result in varying losses of cypermethrin.

## DISCUSSION

The present study shows that washoff of pyrethroids is significantly affected by the specific building material to which it has been applied. Other studies in the published literature have examined washoff from concrete surfaces, turf, and bare soil, so the results from the present study are unique in examining the potential for pyrethroids to wash off from other typical building surfaces. In comparing concrete surface results, Jorgensen and Young 7 reported a 10- to 20-fold higher mass washoff as percentage of applied active ingredient under similar experimental conditions (2.5 cm/h rainfall for 1 h, 24-h delay between application and rainfall) with EC pyrethroid formulations (a.i. esfenvalerate and lambda-cyhalothrin) than the present results. The differences when compared with Cynoff EC may be attributed to several factors, such as differences in formulation, different slopes, different methods of application, and different experimental designs (number of replicates and replicate variability) and scales. Jorgensen and Young used concrete surfaces that were 4 degrees from the horizontal, while the present study used a greater angle (60 degrees from horizontal); therefore, the lower results seen in the present study may be attributed to a shorter retention time of water on the slab surface. Jorgensen and Young pointed out that their method of using a handheld sprayer may be more crude than more precise application methods yet similar to how pest-control operators would apply products. The study concluded that application rates and differences in the active ingredient's chemical properties did not significantly explain the differences seen in total mass washoff between the formulated products. However, both the Jorgensen and Young article and the present study conclude that formulation type can be a contributing factor to total washoff of pyrethroids.

In a more recent study by Jorgensen et al. 8, washoff as percentage of applied mass for EC formulations from concrete surfaces were similar to the results presented in the present study (1.2–2.4% washoff), although different active ingredients and a smaller slope (4 degrees) were used. However, the suspension concentrate formulation showed much greater losses, indicating again that formulation is an important factor in washoff results.

The present study also demonstrated that the surface texture of the material plays a role in the potential transport of pyrethroids in washoff. A similar conclusion was made by Jiang et al. 10 after testing 3 types of surface conditions (stamped, acid wash, and silicone sealant) on concrete slabs. The stamped concrete generally produced less pyrethroids in runoff water than the other surface types. The authors also concluded that the effects of surface conditions on pyrethroid washoff may be increased at a lower rainfall intensity than used in the present study (26.2 mm/h).

## CONCLUSIONS

The present study showed that cypermethrin mass in runoff varied significantly between building material types and formulation. Smoother surfaces produced higher washoff values than textured surfaces, and formulation was found to be significant on surfaces where the roughness was greater. In general, the WP formulation had a higher percentage of washoff overall than the EC formulation. In addition, the present study showed that the amount of water runoff was affected by building material surface and construction. Further work is being conducted to examine the effects of formulation in greater detail. In addition, experiments including larger scales, environmentally weathered material surfaces, and applications under current use practices are needed to determine the relevance of the present results to actual use conditions.

## SUPPLEMENTAL DATA

**Sections S1-S8**

**Tables S1-S4**

**Figure S1-S4.** (294 KB DOCX).

## Supporting Information

All Supplemental Data may be found in the online version of this article.
