# Supplementary material for: Washoff of cypermethrin residues from slabs of external building material surfaces using simulated rainfall
Source: Environ Toxicol Chem. 2013 Oct 15;33(2):302–7. doi: 10.1002/etc.2432 (PMC4253083; doi:10.1002/etc.2432)
Supplement: Supplementary file 1 — Supporting Information. [file etc0033-0302-SD1.doc]

SUPPLEMENTAL DATA

Trask, J.R., C.M. Harbourt, P. Miller, M. Cox, R. Jones, P. Hendley, C. Lam

Washoff of Cypermethrin Residues from Slabs of External Building Material Surfaces Using Simulated Rainfall

Sections S1 – S8

Tables S1 - S4

Figures S1 - S4

*S1* *Test materials*. The test materials selected for the study are outlined in Table S1. Nin-inch wide vinyl wall siding was not available so a vinyl siding product in wider dimensions called soffit (commonly used to cover the underside of exterior building overhangs) was substituted. To help minimize the variability in test slab construction, asphalt and concrete slabs were poured from a single well-mixed truck-load of each material. The concrete, asphalt, and stucco mixtures were poured into custom steel or wooden forms and when set then cut to the proper size; aluminum, vinyl, and wood materials were measured and then cut to size. The asphalt slabs were compacted and distilled water was poured over the slabs to help lower the temperature. The slabs were stored to cool internally to room temperature. Concrete slabs were poured and worked to a smooth surface finish using floats and trowels to mimic wall concrete. Stucco slabs were constructed of a base coat followed by a final top coat, using a float to obtain a non-smooth typical stucco surface.

For all materials, exterior oriented strand board (OSB) plywood (2 cm, nominal 3/4 inch) was used as a dimensionally stable substrate. This backing provided rigidity to the semi-flexible surfaces, but was also needed to attach each slab to the rainfall simulator test stands, which were designed to attach directly to the material and hold it at the correct angle. The final test slab consisted of the building material on top followed by spacer materials and OSB to achieve an overall slab thickness of approximately 3.9 cm (nominal 1.5 inches). The exceptions were asphalt and concrete, which required greater thicknesses (more robust substrate) to duplicate finishes found in typical construction. Final slab thickness for asphalt and concrete were approximately 11.4 (nominal 4.5 in) and 7.6 cm (nominal 3.0 in), respectively.

*S2* *Track sprayer equipment*. A research track sprayer located at the University of Illinois was used for the broadcast application of cypermethrin to the building material slabs. The track sprayer consists of an open topped stainless steel booth with sliding glass doors (2.44 m long by 0.71 m wide). The spray nozzle is attached to the track sprayer arm, which can travel the length of the spray booth (Figure S1). It is an air pressure driven system in which compressed air controls the pneumatic functions of the system. With the system pressurized, the following components can be adjusted: nozzle pressure, speed of the nozzle, and overall system pressure. The pneumatic controls also operate the spray booth doors and the movement of the spray nozzle along the track. The speed of the track sprayer arm is adjusted by moving a dial on the control panel. There are also two air regulators with corresponding dials. The first regulator controls the overall system pressure, which remained at 80 psi for this study, and the second regulator controlled the nozzle pressure, which remained at 30 psi for this study.

For each application run, the track sprayer discharges approximately 35 mL of solution from a 40 mL glass vial to deliver the correct calibrated spray volume and pattern to the 22.86 cm by 60.96 cm test slab area. The height from the slab surface to the nozzle was calibrated along with the correct speed of the track sprayer arm and pressure. Due to the varying thicknesses of slabs, it was necessary to adjust the slab height to maintain the calibrated distance from the slab surface to the spray nozzle. This was achieved using a specially designed hydraulic lift that was placed in the spray booth. Based on calibration trials, TeeJet nozzle model, TP4002E-SS, along with an internal 50 mm mesh screen were used for both test substance applications to deliver the target volume.

*S3* *Test substance application*. Cynoff EC insecticide (approximately 29.5 mL) and Cynoff WP insecticide (approximately 19.2 grams) were mixed with one gallon each of finished tap water in a two-gallon stainless steel spray tank with HDPE tubing manufactured by SOLO and agitated. Applications were made at the recommended maximum label rate (this corresponds to the label rate maximum for a 0.2% solution of cypermethrin applied at a volume approximately 1 L per 10 sq. m or 1 gal per 400 sq. ft. for Cynoff EC and Cynoff WP insecticides). The sprayers had a commercial-grade shut-off valve and were modified with a pressure gage to allow for controlled filling of the 40 mL glass track sprayer vials with the tank mixture. Glass vials were filled by replicate group and capped; therefore, at any given time only one group of vials (ten) was prepared for application. Each glass vial was inverted several times just prior to placing in the track sprayer to ensure the test substance was well mixed. Following each group, the tank mixture was depressurized, the HDPE tubing was drained, and the tank was re-agitated to ensure complete mixing. Following an application, the test slab was removed from the track sprayer and placed in an opaque transport container to dry. The slabs were kept level at all times to keep any of the applied spray mixture from dripping off the slabs. The slabs were allowed to thoroughly air dry before the containers were closed and transported to a separate building for rainfall simulation the following day.

Formulation samples were collected as contingency samples only (e.g., to be analyzed only if there were subsequent questions surrounding applications). A sample of each test substance was extracted from the test substance containers and shipped to FMC for archiving.

Tank mixture samples were collected for each formulation (Table S2). A sample was taken just prior to the first application in the first group to verify, if necessary, the correct mixture of test substance in the spray tank. A second sample was taken following the last application of the last group to verify, if necessary, how well mixed the solution remained during the applications. Tank mixture samples were shipped to the analytical lab for analysis. These samples were segregated from other samples during storage and shipping.

Application monitoring samples (filter paper samples) were collected every fifth application starting with the first application totaling 6 filter paper samples per formulation (e.g., applications 1, 6, and 11). Petri-dish lids, which contained the approximately 15 cm (6 in) diameter filter paper, were placed on the test stand in the track sprayer booth in series and at the same height with the test slab along the length of the sprayer. After application, the sprayed lids were removed and reweighed. Each Petri-dish lid was matched with a Petri-dish bottom and the lid-bottom junction was sealed with electrical tape, wrapped in aluminum foil, and shipped to the laboratory on ice for analysis. Air temperature and relative humidity were monitored through the application process. Temperatures of the test substance during storage and application were monitored using a National Institute of Standards and Technology (NIST) traceable temperature datalogger.

*S4 Sample collection*. The placement of each test stand was based on the dimensions of the simulator test floor and consideration was given to the most uniform rainfall areas within the rainfall simulator test floor, determined during the rainfall simulator verification (Figure S2). Each test stand was leveled upon placement in the rainfall simulator test area and the angle from the vertical checked using a digital protractor and a spare test slab.

The rainfall intensity and duration were set and the simulations began automatically. The rainfall simulator control software was designed, such that the sump pumps and gear motors have an automatic turnoff following the specified rainfall duration. Six discrete rainfall simulation events were performed approximately 24 h following application to the set of test slabs. The position (1 through 11) of each test slab and the field blank in the rainfall simulator was randomly determined using a random number generator assignment in MiniTab Statistical Software (v. 14) [1]. This was done to minimize experimental error and error due to mechanical or system variation across rainfall events (Figure S3). Prior to simulation, each slab was placed on the test stand in the predetermined random position and the sample bottle situated in place for runoff collection. The dry weight of each bottle was recorded. Figure S3 shows the positions of the test stands within the simulator for the first simulation; position assignments for all other simulations are not shown. Air temperature and relative humidity were measured during the simulations. Rain gages, both manual and electronic, were used to verify the rainfall amount and intensity for each event.

Following simulation, slabs were allowed to drain for approximately five minutes. Slabs that had less than one drip in 30 seconds were determined to have ended runoff and were removed prior to the five-minute period. Each sample bottle was weighed, preserved with 0.8 mL 10% formic acid proven to lower the pH to somewhere 5 and 7 depending on the final sample bottle volume, and then reweighed with the addition of the preservative. The sample lids were secured; samples were double bagged, and then packed on wet ice for transport to the analytical laboratory the next day.

To evaluate the stability of cypermethrin in water under transport conditions, a set of three transit stability samples spiked with cypermethrin and a set of three blanks were prepared during the day of simulation. A cypermethrin fortification solution of 10.3 mg/L was prepared by the analytical laboratory and shipped to the test site. Three water samples of 1.5 L each were spiked with 150 µL of the fortification solution to achieve a nominal concentration of 1.03 µg/L and agitated. Each sample bottle was preserved with 0.8mL 10% formic acid (Table S3). Three control samples were also prepared containing 1.5 L of source water. A sample of the source water used to prepare the spray solution (tank mixture) and used in the rainfall simulator was also collected the day of simulation for water characterization analysis and shipped to the laboratory for analysis.

*S5 Sample Analysis*. A Hewlett Packard System GC/MS 5973 MSD was used set with the following conditions: flow at 0.9 mL/min, injection volume of 2 µg/L, oven initial temp of 80 degree Celsius (ºC) with final temperatures of 180 or 305 depending on the ramp rate (40 ºC/min or 5 ºC/min, respectively) , gas type of methane, solvent delay of 16 min, set to low resolution with a plot ion of 207 for cypermethrin and cyfluthrin-methyl-d6 (analytical standard) plot ion of 213. Water samples were measured (100 mL) and extracted by liquid-liquid partition using dichloromethane (DCM). During sample preparation, the prepared sample was shook for 1 min following the addition of DCM. The solution was allowed to separate before draining the DCM and this step was repeated 2 additional times and combining all extracts into the same flask. The DCM was allowed to evaporate and then internal standard solution was used to dissolve all the residues. A 2 µL aliquot of the solution was injected into the GC/MS/NCI. Recoveries seen during method validation were within the acceptable range. From each concentration, 5 spikes were prepared, extracted, analyzed, and results calculated the same as the study samples; the mean recovery was 102% with a relative standard deviation (RSD) of 3%. Laboratory spikes were created using control water or source water from the test site and then spiked with cypermethrin standard solution.

*S6 Simulated rainfall results.* The 3-story rainfall simulator performed consistently with little variability between the six simulation events. The rain gages were placed evenly between the two rows of test slabs with the tipping bucket gage in the center of the simulator. This placement was designed to best understand any variation in rainfall across the simulator area. The mean rainfall amount for the six events was 2.71 cm (SD = 0.07 cm) for a 1 h duration based on the tipping bucket gage whereas the mean rainfall totals from the seven rain gages ranged from 2.61 to 2.77 cm (SD = 0.16 to 0.21 cm). Rain gage 1 (nearest to stand positions 5 and 6) consistently had the lowest rainfall while rain gage 5 (nearest to stand positions 10 and 2) consistently had the highest rainfall. The mean rainfall amount for all events was within 10% of the target rainfall amount (2.54 cm) (Figure S3).

*S7 Water runoff mass results*. The mean masses of water runoff collected from each simulation event by test material are presented in Table S4 and the individual data points are graphically displayed in Figure S4. The water runoff masses collected from the field blanks were not included in the analysis. A split-plot analysis was performed using SAS software (Mixed Models procedure) [2] to determine if there were significant differences between building materials and between the two formulations, as well as between building materials within each level of the formulation and between formulations within each building material. The residuals were normally distributed and appeared to have similar variation across the range of the predicted values so the assumptions for using this type of analysis were met. The analysis showed that the building material was significant but formulation was not significant (*p*=0.67). It was found that there was no interaction between formulation and building material (*p*=0.72). Pairwise comparisons were made using the Tukey adjustment to determine which slabs were significantly different (Table S4). The clean painted and unpainted concrete and dirty painted wood were significantly different (*p*<0.05) from the clean painted and unpainted stucco, clean aluminum siding, clean vinyl siding, and asphalt. Clean unpainted and painted wood were found to be significantly different from clean vinyl siding and asphalt but similar to all other building materials. Asphalt was significantly different from all other building materials. The observed differences in water runoff masses can be primarily attributed to building material rather than other experimental factors such as simulation event. Smooth surfaces may have produced greater losses due to splashing or bouncing of rainfall from the surface. Although the differences in rainfall between positions were reported as minor, these differences may have contributed to the total volume of water in combination with other potential factors. For example, slightly higher rainfall amounts were observed for the western test positions (1-3, 9-11, Figure S3) and surfaces that were randomly positioned in these test positions more often (e.g., clean painted concrete) in combination with their surface texture may have enhanced the volume outcome.

*S8 Transit stability results.* Three samples were taken for transit stability (nominal concentration of 1.03 µg/L) and analyzed to estimate the percent recovery of cypermethrin. Results showed recoveries ranging from 49% to 62% of the expected mass (1.545 µg) per sample with 9%–12% of the spiked chemical being found on the walls of the sample container. The recoveries in these transit spikes were lower than expected and were outside the typical range of acceptable recoveries (70%-120% of nominal). As a result, additional laboratory investigations were undertaken to see if an explanation for these low recoveries could be determined. Similar recoveries were also obtained under laboratory conditions analyzed immediately, showing the low recoveries in the transit samples were related to the presence of acetone in the spiking solution, rather than degradation during the transit time. Furthermore, acceptable recoveries were obtained when formulated product was used to spike some additional samples analyzed after the transit time, suggesting that the lower recoveries appear to be related only to the transit samples spiked with the acetone solution and not to the other samples generated during the study with cypermethrin due to the lack of presence of acetone.

REFERENCES

1. Minitab, Inc. 2008. Minitab Release 16, Multiple Comparison Methods - ID 2011. State College (PA): [cited 2013 May 24]. Available from: http://www.minitab.com/support/answers/answer.aspx?ID=2011

2. SAS Institute. 2012. *Users’s Guide: Statistics, Ver 9*. Cary, NC, USA.

Table S1. Building material characteristics

| Slab Type | Source | Surface Finish | Details |
| --- | --- | --- | --- |
| Clean Unpainted Concrete (CUC) | Blager Concrete, Urbana, IL | Smooth concrete | Steel trowelled to a smooth finish |
| Clean Painted Concrete (CPC) | Blager Concrete, Urbana, IL | Painted smooth concrete | Latex satin finish porch and floor paint applied with a roller |
| Clean Unpainted Stucco (CUS) | Mixture of sand, Portland cement, Type S mortar, distilled water (individual materials obtained from The Home Depot Store #1984) | Pulled trowel textured surface | Smooth top coat followed by a gentle pulling motion that creates a typical stucco surface texture |
| Clean Painted Stucco (CPS) | Mixture of sand, Portland cement, Type S mortar, distilled water (individual materials obtained from The Home Depot Store #1984) | Same as clean unpainted stucco except painted | Latex semi-gloss exterior paint applied with a roller |
| Clean Aluminum Siding (CAL) | The Home Depot Store #1984 (SKU# 093346112445) | White semi-gloss factory finish | Pre-painted aluminum roll-stock |
| Clean Vinyl Siding^1^ (CVL) | Menards, Champaign, IL (SKU#146-1389) | White faux wood finish | Vinyl soffit material |
| Clean Unpainted Wood (CUW) | Armstrong Cash and Carry Lumber Company, Urbana, IL (545 Clear Cedar) | Sanded clear cedar | Clean sanded surface with no knots or imperfections |
| Clean Painted Wood (CPW) | Armstrong Cash and Carry Lumber Company, Urbana, IL (545 Clear Cedar) | Painted over sanded clear cedar | Latex semi-gloss exterior paint applied with a roller |
| Painted Wood with a Dusty Surface (DPW) | Armstrong Cash and Carry Lumber Company, Urbana, IL (545 Clear Cedar) | Same as clean painted wood except the surface was left dusty | After paint drying a California soil was rubbed onto the surface, loose material was removed and the surface misted with water leaving a dusty painted surface |
| Clean Asphalt (ASP) | Open Road Asphalt, Inc. Fairmount, IL (Bituminous Mix- 85BIT4822) | Typical asphalt finish for driveways or roads | Tamped and compacted with a vibratory compactor |

Vinyl wall siding was not available with a nine-inch width. The only vinyl base material available was soffit stock (commonly used to cover the underside of exterior building overhangs). Its profile differed slightly from flat wall siding as it contained two shallow channels to mimic the traditional look of beaded-board wooden soffit.

Table S2. Tank mix solution results

| Sample | Expected Concentration (mg/L) | Measured Concentration (mg/L) | Recovery (%) |
| --- | --- | --- | --- |
| Initial EC Tank Mixture | 2.00 | 1.49 | 75 |
| Final EC Tank Mixture | 2.00 | 1.42 | 71 |
| Initial WP Tank Mixture | 2.00 | 1.72 | 86 |
| Final WP Tank Mixture | 2.00 | 1.68 | 84 |

Table S3. Transit stability sample results

| Transit Sample | Water Volume, (L) | Spiked Mass, (µg) | Cypermethrin in Water (µg) | Cypermethrin in Empty Bottle (µg) | Total Found (µg) | % of Spiked Mass |
| --- | --- | --- | --- | --- | --- | --- |
| 1 | 1.5 | 1.545 | 0.735 | 0.078 | 0.813 | 53 |
| 2 | 1.5 | 1.545 | 0.855 | 0.097 | 0.952 | 62 |
| 3 | 1.5 | 1.545 | 0.675 | 0.084 | 0.759 | 49 |

Table S4. Water runoff masses [g] (mean and standard deviation [SD]) and summary of statistical significance for water runoff across building materials.

| **Building Material** | **Overall Mean Mass *(n*=6)** | **Tukey’s Grouping^a^** |
| --- | --- | --- |
| Clean Unpainted Concrete | 1989 [160] | A |
| Dirty Painted Wood | 1978 [63] | A |
| Clean Painted Concrete | 1940 [122] | A |
| Clean Unpainted Wood | 1826 [159] | A,B |
| Clean Painted Wood | 1793 [128] | A,B |
| Clean Painted Stucco | 1616 [110] | B,C |
| Clean Aluminum Siding | 1600 [160] | B,C |
| Clean Unpainted Stucco | 1593 [181] | B,C |
| Clean Vinyl Siding | 1505 [93] | C |
| Clean Asphalt | 468 [167] | D |

^a^Tukey’s multiple comparison test evaluates pairwise differences between materials. Materials sharing the same letter have mean water runoff mass that are not significantly different. Example: Clean vinyl siding is a “C” which means there is no difference between it and other “C” designations such as clean unpainted stucco; however, asphalt is a “D” which means there is a significant difference between it and clean vinyl siding.


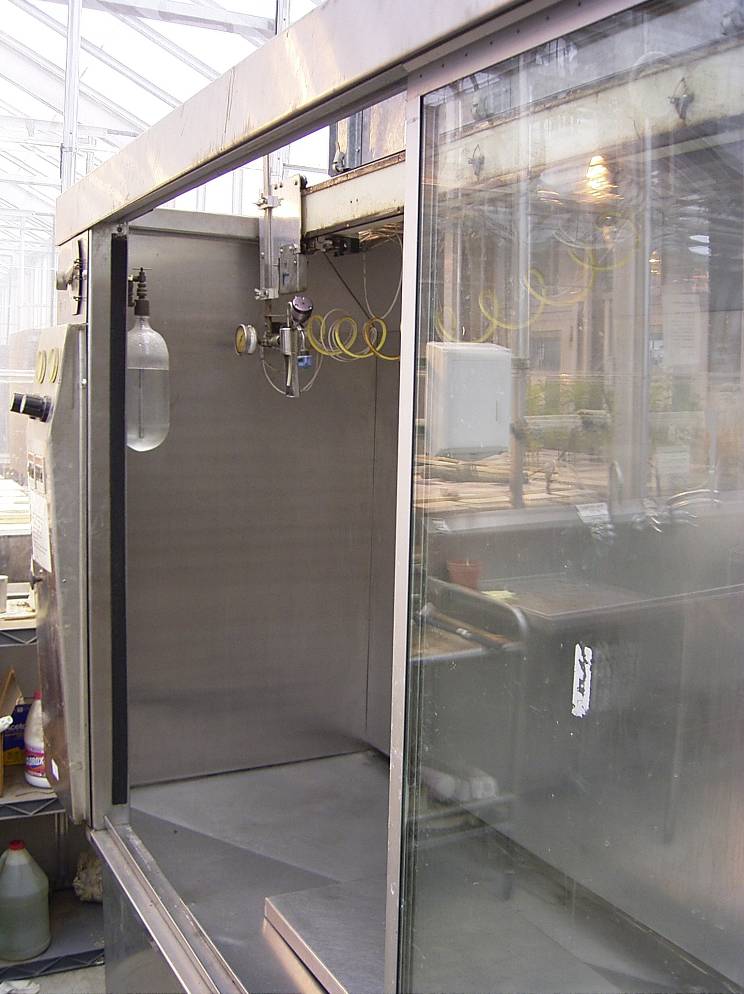


Figure S1. Research track sprayer used for test substance applications to building material slabs. The sprayer arm (upper right corner) holds the 40mL glass vials for test substance application.


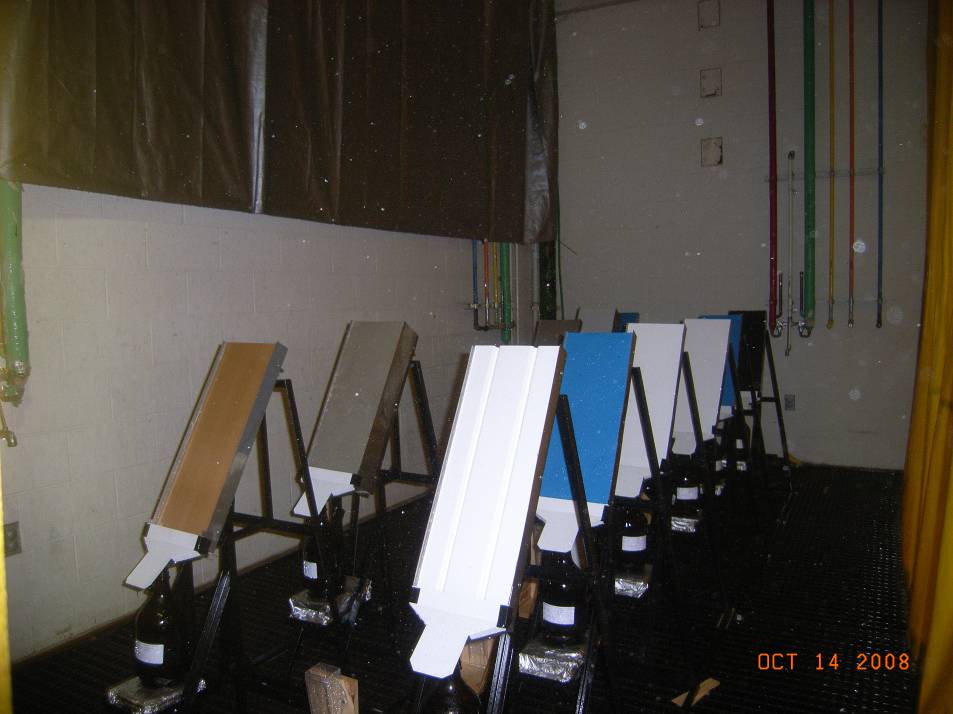


Figure S2. Test stands and collection system for runoff samples from building material slabs.

Figure S3. Example of placement of test stands for rainfall simulations. Positions are labeled 1 to 11 starting in the SW corner of the simulator. 1-ASP=Asphalt, CPW=Clean Painted Wood, CAL=Clean Aluminum Siding, CAL-NA-CS=Field Blank, CPS=Clean Painted Stucco, CVL=Clean Vinyl Siding. 2-Slab identification number (e.g., 01 through 06). 3-Position location in rainfall simulator

Figure S4. Total mass of water runoff from building materials (*n*=3 per building materials/formulation).


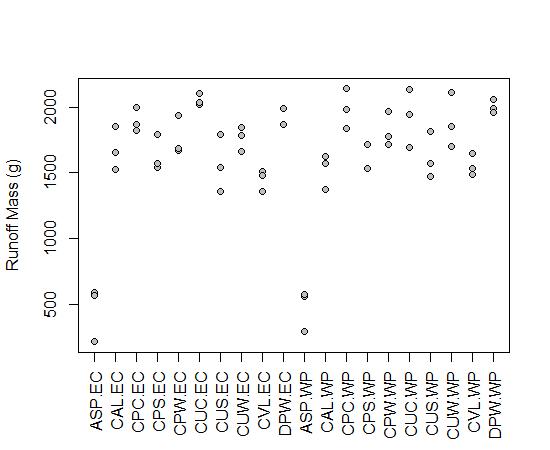


^a^ASP=Clean asphalt, CPW=Clean Painted Wood, CAL=Clean Aluminum Siding, CAL-NA-CS=Field Blank, CPS=Clean Painted Stucco, CVL=Clean Vinyl Siding, DPW=Dirty Painted Wood, CPC=Clean Painted Concrete, CUS=Clean Unpainted Stucco, CUC=Clean Unpainted Concrete, CUW=Clean Unpainted Wood.
